# Identification of interactions between genetic risk scores and dietary patterns for personalized prevention of kidney dysfunction in a population-based cohort

**DOI:** 10.1038/s41387-024-00316-z

**Published:** 2024-08-14

**Authors:** Min-Jae Jang, Li-Juan Tan, Min Young Park, Sangah Shin, Jun-Mo Kim

**Affiliations:** 1 Department of Animal Science and Technology, Chung-Ang University, Gyeonggi-do, 17546 Korea; 2 Department of Food and Nutrition, Chung-Ang University, Gyeonggi-do, 17546 Korea; 3 Department of Molecular Pathobiology, NYU College of Dentistry, New York, USA

**Keywords:** Risk factors, Kidney diseases, Genetics

## Abstract

**Background & Aim:**

Chronic kidney disease (CKD) is a heterogeneous disorder that affects the kidney structure and function. This study investigated the effect of the interaction between genetic factors and dietary pattern on kidney dysfunction in Korean adults.

**Methods:**

Baseline data were obtained from the Ansan and Ansung Study of the Korean Genome and Epidemiology Study involving 8230 participants aged 40–69 years. Kidney dysfunction was defined as an estimated glomerular filtration rate < 90 mL/minute/1.73 m^2^. Genomic DNAs genotyped on the Affymetrix® Genome-Wide Human SNP array 5.0 were isolated from peripheral blood. A genome-wide association study using a generalized linear model was performed on 1,590,162 single-nucleotide polymorphisms (SNPs). To select significant SNPs, the threshold criterion was set at *P*-value < 5 × 10^−^^8^. Linkage disequilibrium clumping was performed based on the R^2^ value, and 94 SNPs had a significant effect. Participants were divided into two groups based on their generic risk score (GRS): the low-GR group had GRS > 0, while the high-GR group had GRS ≤ 0.

**Results:**

Three distinct dietary patterns were extracted, namely, the “prudent pattern,” “flour-based and animal food pattern,” and “white rice pattern,” to analyze the effect of dietary pattern on kidney function. In the “flour-based and animal food pattern,” higher pattern scores were associated with a higher prevalence of kidney dysfunction in both the low and high GR groups (*P* for trend < 0.0001 in the low-, high-GR groups of model 1; 0.0050 and 0.0065 in the low-, high-GR groups of model 2, respectively).

**Conclusions:**

The results highlight a significant association between the ‘flour-based and animal food pattern’ and higher kidney dysfunction prevalence in individuals with both low and high GR. These findings suggest that personalized nutritional interventions based on GR profiles may become the basis for presenting GR-based individual dietary patterns for kidney dysfunction.

## Introduction

Chronic kidney disease (CKD) is a major global health issue affecting millions of people worldwide, and its incidence continues to rise [1]. It is characterized by a gradual loss of kidney function over time, leading to the accumulation of toxic waste products in the body as well as other complications [2]. Kidney dysfunction is a significant risk factor for end-stage renal disease, cardiovascular disease, and premature death [3–5]. Identifying/detecting CKD development at an early stage is important as CKD potentially causes comorbidities, such as cardiovascular disease, as it progresses. The risk of developing cardiovascular disease is considerably high, even during the early stages of CKD [6], and the treatment of patients with cardiovascular risk factors is also known to be effective in attenuating early-stage CKD progression, which causes kidney dysfunction [7]. Consequently, identifying the risk factors for kidney dysfunction is critical for developing effective prevention and treatment strategies.

Certain risk factors for kidney dysfunction, including hypertension, diabetes mellitus, and obesity, have been identified; moreover, increasing evidence suggests that genetic factors may also play a role [8], including several single and polygenic causes [9, 10]. Genome-wide association studies (GWASs) have emerged as a powerful approach toward identifying genetic variants associated with complex diseases. A GWAS is a hypothesis-free study that analyzes millions of common genetic variants across the entire genome to identify those associated with disease risk [11]. It can identify common genetic variants with small-to-moderate effects on disease risk and potentially unravels the biological mechanisms underlying disease development [12]. One important GWAS application is the calculation of genetic risk scores (GRSs), which can estimate an individual’s overall genetic risk (GR) of developing a particular disease [13]. This highlights the importance of GWASs in identifying new GR factors for kidney dysfunction and developing personalized risk-prediction models.

Dietary intake is a well-established modifiable risk factor for CKD, and several dietary patterns have been associated with increased risk [14–17]. The use of dietary patterns instead of individual nutrients or food groups enables the evaluation of the combined effects of multiple nutrients and foods in the diet as well as the impact of their interactions [18, 19]. A healthy dietary pattern is associated with a lower CKD risk, whereas an unhealthy one, such as the Western dietary pattern, is associated with a higher CKD risk [20, 21]. However, genetic contributions to the effects of diet on CKD risk are yet to be fully elucidated. Therefore, investigating the interactions between genetic and dietary factors is essential for enhancing our understanding of the etiology of kidney dysfunction. An interaction analysis of genetic and dietary data can help identify individuals at the highest risk of CKD who may benefit the most from personalized dietary interventions [22]. This method potentially identifies interactions that are typically overlooked when examining each factor individually, providing a more comprehensive understanding of disease etiology. Interaction analyses require large sample sizes, such as a cohort, to detect significant interactions, and this can present a challenge for diseases [23].

Therefore, this study aimed to propose an appropriate GRS-based dietary pattern for kidney dysfunction in the Korean population. In addition, it sought to identify new GR factors and calculate GRSs for kidney dysfunction in a large, population-based cohort in Korea by cross-sectional analysis. Furthermore, it also investigated dietary patterns as well as the effect of the interactions between genetic and dietary factors on kidney dysfunction risk using both association and interaction analyses. This study’s findings may provide new insights into the etiology of kidney dysfunction in the Korean population and help develop personalized dietary recommendations for individuals at high risk of kidney dysfunction.

## Materials and methods

### Study design and population

**Fig. 1:**
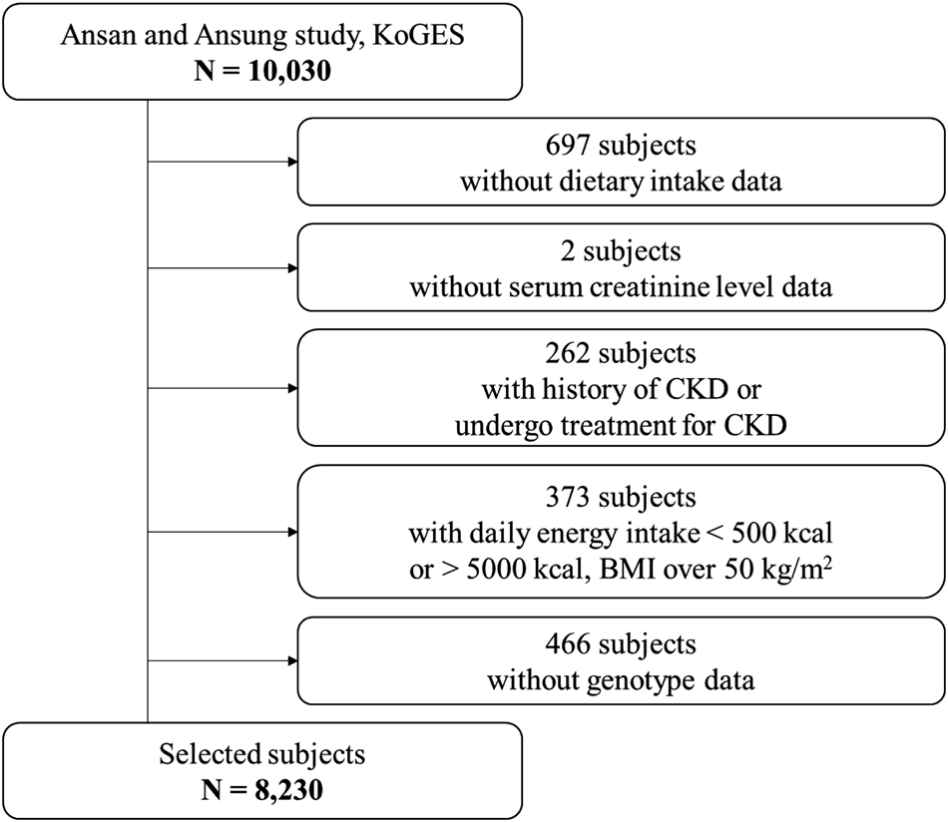
Flow chart of the participants for the kidney dysfunction study. Participants were selected from the Ansan and Ansung cohort of the KoGES study, starting with an initial pool of 10,030 individuals. Exclusions were made for missing dietary intake or serum creatinine data, history of CKD, extreme energy intake or BMI, and missing genotype data, resulting in a final sample of 8230 participants.

Participants were recruited from the Ansan and Ansung Study of the Korean Genome and Epidemiology Study (KoGES) conducted from 2001 to 2002 to investigate common chronic diseases in Koreans by Korea Disease Control and Prevention Agency (KDCA). A total of 8230 Korean adults aged 40–69 years, including men (47.9%) and women (52.1%), were enrolled, and whole-genome single-nucleotide polymorphism (SNP) genotyping was performed. This study was performed to investigate the environmental and genetic causes of common diseases associated with kidney dysfunction. From the 10,030 participants, those without dietary intake and serum creatinine level data (*n* = 697) as well as those with serum creatinine levels outside the three-standard-deviation range from the mean value (*n* = 2) were excluded. Moreover, participants on CKD medication (*n* = 262), those with abnormal energy intake (< 500 or > 5000 kcal), those with a body mass index (BMI) > 50 kg/m^2^ (*n* = 373), and those with missing genotypic data (*n* = 466) were also excluded. In addition, participants with a history of CKD were excluded during the selection process. Thus, 8230 participants were finally included (Fig. 1). The data-use protocol was adopted from the KoGES (4851–302) and approved by the National Research Institute of Health, Centers for Disease Control and Prevention, Ministry of Health and Welfare, Republic of Korea.

### Definition of kidney dysfunction

CKD is classified into stages 1–5 based on the estimated glomerular filtration rate (eGFR), and this classification can evaluate kidney function, with the degree of kidney damage and decrease in function varying depending on the stage [24]. Moderate CKD is diagnosed based on the stage when the eGFR level is < 60 mL/min/1.73 m^2^. This study analyzed genetic and dietary effects on early stage of CKD, including stage 2, wherein kidney function begins to deteriorate. Stage 2 CKD is defined as an eGFR < 90 mL/min/1.73 m^2^ [25]. In this study, we specifically defined kidney dysfunction as an eGFR < 90 mL/min/1.73 m^2^, marking the onset of potential early-stage CKD [26]. We focused on the decline in eGFR as an indicator of kidney dysfunction and aim to explore the onset and progression of kidney dysfunction starting from these early stages, in order to identify potential early indicators and interventions for CKD. Serum creatinine levels were measured colorimetrically (Hitachi Automatic Analyzer 7600), and the eGFR was calculated using the Chronic Kidney Disease Epidemiology Collaboration equation [27]

For females, if the serum creatinine concentration (μmol/L [mg/dL]) was ≤ 62 (≤ 0.7),

$${{\rm{e}}{\rm{GFR}}=144\times ({\rm{Scr}}/0.7)}^{{{\mbox{-}}}0.329}\times {(0.993)}^{{\rm{age}}}$$

For females, if the serum creatinine concentration (μmol/L [mg/dL]) was > 62 (> 0.7),

$${{\rm{e}}{\rm{GFR}}=144\times ({\rm{Scr}}/0.7)}^{{{\mbox{-}}}1.209}\times {(0.993)}^{{\rm{age}}}$$

For males, if the serum creatinine concentration (μmol/L [mg/dL]) was ≤ 80 (≤ 0.9),

$${{\rm{e}}{\rm{GFR}}=141\times ({\rm{Scr}}/0.9)}^{{{\mbox{-}}}0.411}{\times (0.993)}^{{\rm{age}}}$$

For males, if the serum creatinine concentration (μmol/L [mg/dL]) was > 80 (> 0.9),

$${{\rm{e}}{\rm{GFR}}=141\times ({\rm{Scr}}/0.9)}^{{{\mbox{-}}}1.209}{\times (0.993)}^{{\rm{age}}}$$

### GRS estimation

Genomic DNA was extracted from peripheral leukocytes drawn from Ansung and Ansan cohort participants. Genotyping was performed using Affymetrix® Genome-Wide Human SNP Array 5.0 (Affymetrix, Inc., Santa Clara, CA, USA). Through the KDCA, imputed data containing whole-genome information for the cohort was obtained. Markers were filtered for call rate, minor allele frequency (MAF), and the Hardy–Weinberg equilibrium by referring to the criteria applied in a previous study [28]. SNP imputation was performed using the IMPUTE program [29]. The imputation was based on NCBI build 35 and dbSNP build 126, and it initially included 90 individuals from JPT and CHB founders in HapMap as a reference (HapMap release 22). After removing SNPs with a MAF < 0.01 and an SNP missing rate > 0.05, Ansung and Ansan cohort SNPs were combined with 1.5 million imputed SNPs for association analyses with the selected quantitative trait.

A GWAS of the interaction between SNPs and kidney dysfunction was performed using the GCTA program tested by generalized linear association analysis after adjusting for sex as a fixed effect and age, BMI, hemoglobin A1c, and blood pressure as covariates using 1,590,162 SNPs [30]. The threshold criterion was set at *P*-value < 5 × 10^–8^ for SNPs from the GWAS. Linkage disequilibrium (LD) clumping and LD analysis were performed using the causal variants identified in the associated regions (CAVIAR) program to identify causal SNPs located in trait-associated regions [31]. SNP locations and nomenclature were defined using Ensembl 54. Each participant’s GRS was calculated using the 94 most strongly associated SNPs according to the following model [1]:

$${\rm{GRS}}=\mathop{\sum }\limits_{i=1}^{n}{{risk\; allele\; in\; SNP}}_{i}\times {\beta }_{i}$$

The beta values represent the effective size of increasing the GFR in each SNP. Therefore, a high or low GRS is associated with a high or low risk of developing low GFR levels, respectively. For the analysis, the low-GR group included participants with a GRS > 0, whereas the high-GR group included those with a GRS ≤ 0.

### Covariates

This population’s general attributes were obtained via questionnaires as well as anthropometric and clinical measurements, such as height, weight, and BMI (weight [kg] ÷ height^2^ [m^2^]). Demographic data were categorized based on two cities (Ansan and Ansung); household income level (expressed as monthly income in Korean won [KRW], where 1 million KRW is approximately 750 United States dollars), which was categorized into three brackets (< 1 million, < 3 million, and > 3 million KRW); drinking habits (current drinker, past drinker, or non-drinker); smoking habits (current smoker, past smoker, or non-smoker); and daily physical activity (none, < 30 min, 30–60 min, 60–90 min, 90 min to 2 h, 2–3 h, 3–4 h, 4–5 h, or > 5 h). Physical activity was measured in metabolic equivalents (MET) [32], and based on the International Physical Activity Questionnaire (IPAQ), and participants were categorized into three groups based on the International Physical Activity Questionnaire (IPAQ): “low,” “moderate,” and “high” [33]. “High activity” individuals met specific criteria, which included engaging in vigorous-intensity activity on ≥ 3 days per week, resulting in a minimum of 1500 MET-min per week, or participating in ≥ 7 days of various activities, achieving a minimum of 3000 MET-min per week. The “moderate activity” group comprised individuals who met any of the following criteria: vigorous-intensity activity for ≥ 20 min per day on ≥ 3 days per week, engaging in moderate-intensity activity and/or walking for ≥ 30 min per day on ≥ 5 days per week, or participating in any combination of walking, moderate-intensity activity, and/or vigorous-intensity activity for ≥ 5 days per week, resulting in a total physical activity of ≥ 600 MET-min per week. Individuals who did not meet the criteria outlined above were classified in the “low activity” group.

### Dietary assessment and dietary pattern analysis

Dietary intake was assessed using a food-frequency questionnaire to extract information regarding the average consumption frequencies and serving sizes of 103 food items [34]. For factor extraction, principal component analysis was applied, and for factor rotation, varimax rotation was employed. We identified and selected the initial 22 factors that had eigenvalues ≥ 1.4 (Supplementary Table 1). To construct the final factors, we specifically considered components that effectively accounted for a significant portion of the variance within each factor and exhibited factor loadings ≥ 0.3. By adhering to these criteria, we established a total of three dietary patterns and assigned them names based on the characteristic food groups they encompassed. To explore the relationship between these dietary patterns and kidney dysfunction, we divided the factor scores into tertiles, denoted as T1, T2, and T3. We calculated representative nutrient intake levels based on tertiles for each dietary pattern (Supplementary Table 3).

### Statistical analysis

Statistical analyses were conducted using SAS software (version 9.4; SAS Institute, Inc.), and statistical significance was set at *P*-value < 0.05. Categorical data are expressed as the number of participants (%), while continuous data are presented as the mean ± standard deviation. Multivariable-adjusted logistic regression and joint interaction analyses were employed to determine the odds ratios (ORs) and 95% confidence intervals (CIs) of kidney dysfunction across the tertiles of each dietary factor and two GR groups, with adjustments made for covariates. Regression modeling was performed to analyze *P*-values for trends.

## Results

### Characteristics of the study population

The participants’ general characteristics based on the KoGES Ansung and Ansan cohort are described in Table 1. Participants were categorized into two GR groups. Those with high GR tended to have a lower eGFR (*P*-value < 0.0001) and household income (*P*-value < 0.0001) and were more likely to be current alcohol drinkers (*P* = 0.0034) and smokers (*P* = 0.0185).

**Table 1:** Basic characteristics according to genetic risk of kidney dysfunction among Korean adults.

	GR low	GR high	*P*-value
*N*	3959	4271	
Men (%)	1769 (48.10)	2175 (51.90)	< 0.0001
Age (year)	52.57 ± 8.90	51.68 ± 8.87	< 0.0001
BMI (kg/m^2^)	24.62 ± 3.21	24.57 ± 3.04	0.4421
eGFR	93.04 ± 17.04	88.82 ± 16.83	< 0.0001
Household income			
<1,000,000 KRW	1482 (37.43)	1316 (30.81)	< 0.0001
<3,000,000 KRW	1816 (45.87)	2044 (47.86)	
≥3,000,000 KRW	595 (15.03)	864 (20.23)	
Drinking status			
Current alcohol drinker	1805 (45.59)	2110 (49.40)	0.0034
Past alcohol drinker	262 (6.62)	262 (6.13)	
Nondrinker	1897 (47.92)	1888 (44.21)	
Smoking status			
Current smoker	962 (24.30)	1125 (26.34)	0.0185
Past smoker	596 (15.05)	702 (16.44)	
Nonsmoker	2371 (59.89)	2405 (56.31)	
Physical activity			
Low	225 (5.68)	261 (6.11)	0.0188
Moderate	756 (19.10)	913 (21.38)	
High	2978 (75.22)	3097 (72.51)	

*GR* Genetic risk, *BMI* Body mass index, *eGFR* estimated glomerular filtration rate, *KRW* Korean won.

### GWAS of kidney dysfunction

**Fig. 2:**
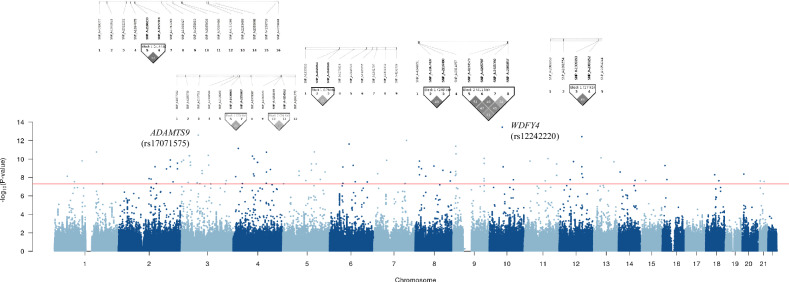
Manhattan plot of the genome-wide association analysis results of eGFR levels. The GWAS results for eGFR levels are shown, with –log_10_ *P*-values for SNPs plotted across all chromosomes. The red horizontal line indicates the genome-wide significance threshold of *P*-value < 5 × 10^–8^.

**Fig. 3:**
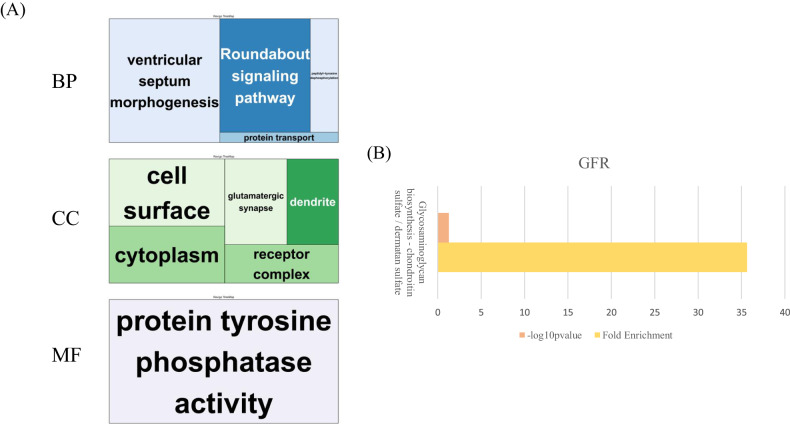
Functional analysis of significant genes. **A** Gene Ontology treemaps of biological processes, cell components, and molecular function according to significant genes. **B** Bar graph of Kyoto Encyclopedia of Genes and Genomes (KEGG) pathways.

A total of 1,590,162 SNPs were utilized in the GWAS. The highest number of SNPs was found on human chromosome 1, while the lowest was observed on chromosome 19. On average, the SNPs located on each chromosome were approximately 1856.0 base pairs apart. Among the chromosomes, chromosome 19 had the widest interval between SNPs, while chromosome 6 had the narrowest. These details are summarized in Table 2. A GWAS was performed to identify significant loci related to kidney dysfunction. Quantile-quantile (Q-Q) plot and Manhattan plot for the genetic association were presented to visualize the distribution of observed *P*-values against the expected values (Fig. 2 and Supplementary Fig. 1). Among the 86,030 significant SNPs (*P*-value < 0.05), 109 were selected based on a *P*-value < 5 × 10^−^^8^. We identified 94 SNPs after screening for crucial effects on the GFR and calculted GRS on each participant. The odds ratio according to GR group is presented in Suppplementary Table 4. These SNPs were annotated on twenty chromosomes. Among them, the most significant SNPs with the highest absolute beta values were rs17071575 on chromosome 3 (beta value: −7.3) and rs12242220 on chromosome 10 (beta value: −7.6). These SNPs were annotated on the *ADAMTS9* and *WDFY4* genes. *ADAMTS9* has been implicated in the pathogenesis of juvenile nephronophthisis and nephronophthisis, two pathological conditions [35]. Relevant *ADAMTS9*-associated biological pathways include those related to O-glycosylation of proteins and protein metabolism. Moreover, the function of *WDFY4* is associated with its involvement in various cellular processes, including autophagy, endocytosis, and intracellular trafficking. Gene ontology (GO) enrichment analyses were performed with significant genes based on the GWAS results (Fig. 3). In terms of the biological process, ventricular septum morphogenesis was significantly enriched. Regarding cellular composition, cell surfaces were enriched, and tyrosine phosphatase activity was significantly enriched in molecular function. Moreover, enriched pathways for annotated genes were determined using the Kyoto Encyclopedia of Genes and Genomes database. In particular, the glycosaminoglycan biosynthesis–chondroitin sulfate/dermatan sulfate pathway was enriched.

**Table 2:** The numbers of available SNPs in the Ansung and Ansan cohort and average interval distances between adjacent SNPs.

Chromosome	Numbers of SNPs	Average interval (bp)	Standard deviation (bp)	Total distance (bp)
1	124,121	1985.1	60,894.7	246,391,884
2	142,070	1708.2	11,386.2	242,679,861
3	111,287	1790.7	14,436.3	199,285,064
4	101,257	1887.4	12,004.2	191,109,993
5	108,867	1658.6	11,660.6	180,569,964
6	120,023	1421.9	9528.9	170,659,819
7	88,026	1802.5	13,295.7	158,663,166
8	95,470	1530.5	12,331.7	146,112,942
9	77,035	1816.8	94,118.5	139,952,233
10	89,719	1506.9	11,001.6	135,200,383
11	84,757	1584.0	11,582.3	134,256,672
12	77,661	1702.7	6560.0	132,229,515
13	65,544	1465.1	3539.7	96,027,786
14	51,388	1693.0	4329.3	86,998,491
15	42,947	1903.9	8552.0	81,763,030
16	39,351	2253.2	52,705.0	88,662,122
17	30,603	2568.0	8240.5	78,586,573
18	46,858	1621.2	8674.0	75,966,267
19	17,914	3549.4	61,097.3	63,580,903
20	36,213	1721.7	12,417.9	62,344,784
21	21,109	1747.9	22,825.8	36,895,145
22	17,942	1912.9	6455.9	34,319,536
	Total: 1,590,162	Average: 1856.0	Average: 20,801.7	Total: 2,782,256,133

*SNP* Single-nucleotide polymorphism, *bp* base pair.

### The interactive effects of dietary patterns and GR on kidney dysfunction prevalence

**Fig. 4:**
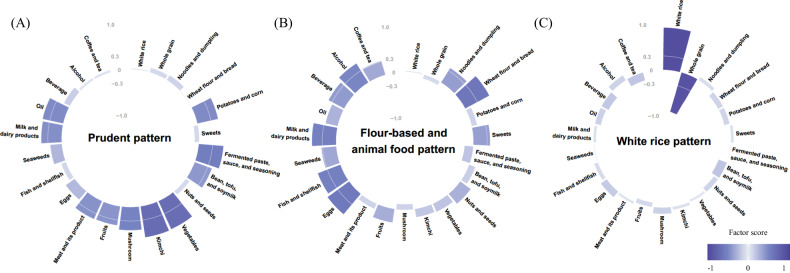
Factor loading for major dietary patterns identified via factor analysis. **A** “Prudent pattern”, **B** “Flour-based and animal food pattern”, **C** “White rice pattern”. The dietary patterns were named based on the top food groups that had the highest factor-loading values.

Three major dietary patterns were identified from the factor analysis, and they were named after foods or food groups with high factor-loading values (Fig. 4, Supplementary Table 2). The intake of energy, carbohydrates, proteins, and lipids increased with higher adherence to prudent, flour-based, and animal food patterns, while it decreased in the white rice pattern (all *P*-value < 0.0001) (Supplementary Table 3) and odds ratio for each dietary pattern is shown in Supplementary Table 5. The “prudent pattern” was characterized by a high consumption of vegetables, kimchi, fermented paste, sauce, seasoning, and mushroom. The “flour-based and animal food pattern” was typified by a high consumption of wheat flour and bread, eggs, fish, and shellfish. The “white rice pattern” featured highly positive factor loadings for white rice and negative factor loadings for whole grains.

The ORs and 95% CIs of kidney dysfunction across the interactions of dietary pattern tertiles with eGFR levels are presented in Table 3. The kidney dysfunction prevalence was analyzed based on alterations in the dietary pattern score at each GR level, and the lowest tertile (T1) at each GR level was used as the reference group. First, the highest “prudent pattern” tertile exhibited a significantly decreased kidney dysfunction prevalence compared with T1 in model 1 of the high-GR group (*P* for trend = 0.0332); however, this association attenuated after adjustments in model 2 (*P* for trend = 0.2152). Participants in the T2 and T3 “prudent pattern” tertiles of the low-GR group displayed a borderline decrease; nevertheless, it was not significant compared with that in T1 (*P* for trend = 0.0782 and 0.0840 in models 1 and 2, respectively). In the “flour-based and animal food pattern,” a higher pattern score was associated with a higher OR in the low- and high-GR groups of both models 1 and 2 (*P* for trend < 0.0001 in the low- and high-GR groups of model 1; *P* for trend = 0.0050 and 0.0065 in the low- and high-GR groups of model 2, respectively). In contrast, in the “white rice pattern” of the high-GR group, participants with the highest pattern scores had a significantly decreased kidney dysfunction prevalence compared with those in T1 of model 1 (*P* for trend < 0.0001); nonetheless, the association weakened after adjustments in model 2, and no significant interactions were observed (*P* for trend = 0.0775). Low-GR participants with T2 and T3 pattern scores exhibited an increased prevalence of kidney dysfunction; however, it was not significant compared with that of those in T1 (*P* for trend = 0.9413 and 0.0894 in models 1 and 2, respectively).

**Table 3:** The odds ratios of kidney dysfunction according to the dietary pattern score tertiles stratified by the GRS among Korean adults.

		Tertile of Each Pattern Score			
		T1	T2	T3	
		OR	OR (95% CI)	OR (95% CI)	*p* for trend
**Prudent pattern**					
**model 1**	**GR low**	543/1319	557/1320	505/1320	
		1.00 (ref.)	1.041 (0.889–1.219)	0.880 (0.750–1.031)	0.0782
	**GR high**	756/1423	779/1424	719/1424	
		1.00 (ref.)	1.053 (0.907–1.223)	0.864(0.744–1.003)	0.0332
	***p*** **for interaction**	0.808			
**model 2**	**GR low**	1.00 (ref.)	1.015 (0.861–1.196)	0.864 (0.723–1.033)	0.0840
	**GR high**	1.00 (ref.)	1.015 (0.868–1.186)	0.904 (0.763–1.072)	0.2152
	***p*** **for interaction**	0.877			
**Flour-based and animal food pattern**					
**model 1**	**GR low**	548/1319	508/1320	549/1320	
		1.00 (ref.)	1.115 (0.947–1.313)	1.478 (1.247–1.753)	< 0.0001
	**GR high**	746/1423	724/1424	784/1424	
		1.00 (ref.)	1.125 (0.964–1.313)	1.456 (1.241–1.709)	< 0.0001
	***p*** **for interaction**	0.414			
**model 2**	**GR low**	1.00 (ref.)	0.996 (0.838–1.185)	1.306 (1.068-1.598)	0.0050
	**GR high**	1.00 (ref.)	0.999 (0.847–1.177)	1.283 (1.061–1.552)	0.0065
	***p*** **for interaction**	0.703			
**White rice pattern**					
**model 1**	**GR low**	539/1319	528/1320	538/1320	
		1.00 (ref.)	1.015 (0.866–1.189)	1.005 (0.857–1.177)	0.9413
	**GR high**	812/1423	746/1424	696/1424	
		1.00 (ref.)	0.837 (0.721-0.973)	0.720 (0.620–0.837)	< 0.0001
	***p*** **for interaction**	0.003			
**model 2**	**GR low**	1.00 (ref.)	1.057 (0.897–1.246)	1.159 (0.981–1.368)	0.0894
	**GR high**	1.00 (ref.)	0.913 (0.781–1.067)	0.868 (0.741–1.017)	0.0775
	***p*** **for interaction**	0.011			

model 1: adjusted for sex (men and women) and age (continuous).

model 2: adjusted for sex, age, BMI, energy intake, household income level, alcohol drinking status, smoking status, education level, physical activity.

*GRS* Genetic risk score, *OR* Odds ratio, *GR* Genetic risk, *BMI* Body mass index.

In the joint-effect model, with a low dietary pattern score and low GR as reference points, a high “prudent pattern” score and low GR were associated with the lowest prevalence of kidney dysfunction (OR: 0.899, 95% CI: 0.758–1.065, *P* for trend = 0.0840). The highest prevalence was observed in individuals with a low pattern score and high GR (OR: 1.608, 95% CI: 1.373–1.883, *P* for trend = 0.2152). In the “flour-based and animal food pattern,” the lowest prevalence was observed in low-GR individuals with T2 pattern scores (OR: 0.971 95%, CI: 0.82–1.148, *P* for trend = 0.0050), whereas the highest prevalence was noted in high-GR individuals with high pattern scores (OR: 2.061, 95% CI: 1.719–2.471, *P* for trend = 0.0065). In addition, in the “white rice pattern,” the lowest prevalence was observed in those with low pattern scores and low GR (reference group), while the highest prevalence was noted in those with low pattern scores and high GR (OR: 1.828, 95% CI: 1.560–2.142, *P* for trend 0.0775) (Table 4).

**Table 4:** The odds ratios of kidney dysfunction according to the joint categories of each dietary pattern score and the GRS among Korean adults.

	Tertile of Each Pattern Score			
	T1	T2	T3	
	OR (95% CI)	OR (95% CI)	OR (95% CI)	*p* for trend
**Prudent pattern**				
**GR low**	543/1319	557/1320	505/1320	
	1.00 (ref.)	1.025 (0.872–1.206)	0.899 (0.758–1.065)	0.0840
**GR high**	756/1423	779/1424	719/1424	
	1.608 (1.373-1.883)	1.620 (1.381–1.899)	1.420 (1.203–1.676)	0.2152
**Flour-based and animal food pattern**				
**GR low**	548/1319	508/1320	549/1320	
	1.00 (ref.)	0.971 (0.822–1.148)	1.274 (1.063–1.527)	0.0050
**GR high**	746/1423	724/1424	784/1424	
	1.548 (1.322–1.813)	1.595 (1.351–1.884)	2.061 (1.719–2.471)	0.0065
**White rice pattern**				
**GR low**	539/1319	528/1320	538/1320	
	1.00 (ref.)	1.047 (0.890–1.231)	1.161 (0.986–1.367)	0.0894
**GR high**	812/1423	746/1424	696/1424	
	1.828 (1.560–2.142)	1.676 (1.431–1.963)	1.588 (1.355–1.861)	0.0775

Adjusted for sex, age, BMI, energy intake, household income level, alcohol drinking status, smoking status, education level, physical activity.

*GRS* Genetic risk score, *OR* Odds ratio, *GR* Genetic risk, *BMI* Body mass index.

## Discussion

Kidney dysfunction is a global public health concern with increasing prevalence, leading to complications like cardiovascular disease and end-stage kidney disease. This study investigates how genetic risk scores (GRSs) interact with dietary choices to influence kidney dysfunction risk in a Korean population, aiming to provide personalized dietary recommendations for prevention.

### Genetic-variant identification and gene function associated with kidney dysfunction

GWAS analysis of genetic influence revealed that the final 94 SNPs were significantly related to renal function deterioration. Regarding genetic influence, the disease was not affected by a specific gene or loci, but by several genes.

Specifically, rs17071575 and rs12242220 (located on the *ADAMTS9* and *WDFY4* genes) on chromosomes 3 and 10, respectively, were the most significant SNPs with the highest absolute beta values in relation to kidney dysfunction. These findings are consistent with those of previous studies that also identified a link between these genes and kidney disease. Numerous studies have suggested that the *WDFY4* gene plays a significant role in kidney dysfunction development as well as hypertension, which is a common risk factor for kidney dysfunction [36], and exerts a significant effect on kidney function and immune response [37, 38]. Moreover, the *WDFY4* gene is reportedly involved in regulating the glomerular filtration barrier size in the kidneys, suggesting its crucial role in renal physiology [39, 40]. Furthermore, the *ADAMTS9* gene is potentially involved in kidney dysfunction development. Several genetic association studies have found a significant association between *ADAMTS9* polymorphisms and kidney function decline in different populations, including European, Chinese, and Japanese individuals [41, 42]. Furthermore, a GWAS identified *ADAMTS9* as a novel genetic locus associated with kidney function in the general population [43]. *ADAMTS9* reportedly regulates the extracellular matrix and cell signaling pathways, which serve crucial roles in renal physiology [44]. The *ADAMTS9* gene has also been linked to hypertension, a common risk factor for kidney disease. These findings suggest that the *WDFY4* and *ADAMTS9* genes potentially play an essential role in kidney dysfunction pathogenesis and highlight the requirement for further investigation to elucidate the mechanisms underlying this relationship.

### Kidney dysfunction prevalence according to dietary pattern

As several treatments for kidney-related diseases are diet-based, specific nutrient intake is also important; however, the strong association between the main dietary pattern and kidney dysfunction prevalence in Koreans bears significance. Koreans have been found to exhibit three main consumption patterns: the “prudent pattern,” “flour-based animal food pattern,” and “white rice pattern.”

The “prudent dietary pattern” is reportedly beneficial for people with kidney dysfunction in several ways. Adherence to a healthy dietary pattern, such as high fruit, vegetable, whole-grain, and low-fat dairy product intakes, has been associated with a lower risk of developing CKD in women [20]. Additionally, limiting dietary phosphorus and protein intakes by reducing the consumption of animal protein-source foods, such as meat, may help decelerate CKD progression [45]. A low-phosphorus diet reportedly alleviates CKD by reducing the levels of serum fibroblast growth factor 23, which is associated with CKD development and progression [46]. Concerning protein intake, debate regarding the optimal intake for people with kidney dysfunction persists. A low-protein diet (0.6 g of protein per kilogram of body weight per day) has been found to attenuate CKD progression in patients with moderate-to-severe kidney dysfunction [47]. However, other studies have suggested that a higher protein intake may be safe and even beneficial for some patients with kidney dysfunction. Evidently, the “prudent dietary pattern” can be helpful for people with kidney dysfunction.

The “flour-based animal food pattern” typically refers to a diet that is high in refined grains and processed meats, which are both associated with an increased risk of kidney dysfunction. Previous research has revealed an association between a higher intake of processed meats and a greater risk of developing CKD [48]. Excessive protein intake, particularly from animal sources, is potentially harmful to the kidneys and may contribute to CKD development or progression [49]. Additionally, a high sodium intake, which is common in flour- and animal-based foods, can increase blood pressure and exacerbate fluid retention, further compromising kidney function [50]. Previous studies have found that individuals with CKD who consume high-protein diets experience a faster decline in kidney function than those who consume low-protein diets [47]. Moreover, a diet high in animal protein has been associated with an increased risk of kidney stone formation [51]. Conversely, plant-based protein sources, such as legumes and tofu, are reportedly beneficial to kidney function. A low-protein, high-fiber diet that included plant-based protein sources was found to improve kidney function in individuals with CKD [52]. Therefore, the “flour-based animal food pattern” may not be the optimal dietary approach for individuals with kidney dysfunction. Instead, a diet that is low in animal-based protein and high in plant-based protein and fiber may be more beneficial for supporting kidney function.

Previous studies have established an association between the “white rice pattern,” characterized by a high intake of white rice, and increased CKD. Positive correlations exist between the frequency of white rice consumption and CKD incidence in Korean adults. A high intake of white rice potentially increases kidney dysfunction risk in Koreans. The high glycemic index (GI) of white rice has been suggested as a potential mechanism underlying the association between white rice consumption and kidney dysfunction. High GI foods potentially lead to a rapid increase in blood glucose and insulin levels, which may contribute to insulin resistance, inflammation, and oxidative stress, all of which are associated with kidney dysfunction [53].

### Interactive effects of GR and dietary patterns on kidney dysfunction prevalence

Our study provides evidence that the prevalence of kidney dysfunction may be significantly influenced by the interactive effects of GR and dietary patterns. This finding is consistent with those of previous studies that have demonstrated how disease prevalence can be influenced by varying dietary intakes, depending on GRSs. For instance, higher-GR individuals who consume fried foods are more likely to have an increased BMI [54], while those with a higher GR for obesity who follow a healthy diet have a lower risk of weight gain [55]. This highlights the importance of personalized diets in reducing disease prevalence. Customized dietary interventions potentially reduce disease prevalence among individuals with specific GR profiles. It emphasizes the potential advantages of modifying dietary patterns based on an individual’s GR profile. This enables personalized nutritional interventions that enhance health outcomes in the prevention and management of kidney dysfunction. Our research has limitations as it focused on a specific population, which may affect result variations in different ethnic or demographic groups. Additionally, the dietary data collected were self-reported, which may introduce bias and inaccuracies. Furthermore, dietary patterns can vary over time, and our study captured only a snapshot of participants’ diets. However, recognizing these limitations, our study underscores the importance of considering dietary patterns in conjunction with genetic risk factors for personalized nutritional interventions that enhance health outcomes in the prevention and management of kidney dysfunction. Future studies should replicate our findings in diverse populations to improve generalizability. Exploring molecular pathways and gene-diet interactions can further enhance our understanding and allow for customized prevention strategies, ultimately transforming public health interventions for more effective kidney disease prevention.

## Conclusion

Our study emphasizes the significant interaction between dietary patterns and genetic susceptibility, and kidney dysfunction. This study’s findings have significant implications for public health interventions targeted at mitigating the impact of kidney dysfunction. Encouraging healthier dietary patterns, particularly in individuals with a significant genetic predisposition, is a potentially effective approach toward preventing or managing kidney dysfunction. Additionally, evaluating genetic susceptibility can assist in the identification of individuals who may benefit the most from targeted dietary interventions.

### Supplementary information

Supplemantary figure 1

Supplementary table 1

Supplementary table 2

Supplementary table 3

Supplementary table 4

Supplementary table 5

## Data Availability

The datasets used and/or analyzed during the current study are owned by a third-party organization (The Korean Genome and Epidemiology Study–Ansan and Ansung Study, KoGES; 4851-302). These data have been availed by an online sharing service under the permission of the Division of Epidemiology and Health Index, Korea Centers for Disease Control and Prevention (KCDC). Further details are available in English on the following website: http://www.nih.go.kr/contents.es?mid=a50401010400#1.
