# Supplementary figures and images for: Identification of interactions between genetic risk scores and dietary patterns for personalized prevention of kidney dysfunction in a population-based cohort
Source: Nutr Diabetes. 2024 Aug 14;14:62. doi: 10.1038/s41387-024-00316-z (PMC11325018; doi:10.1038/s41387-024-00316-z)

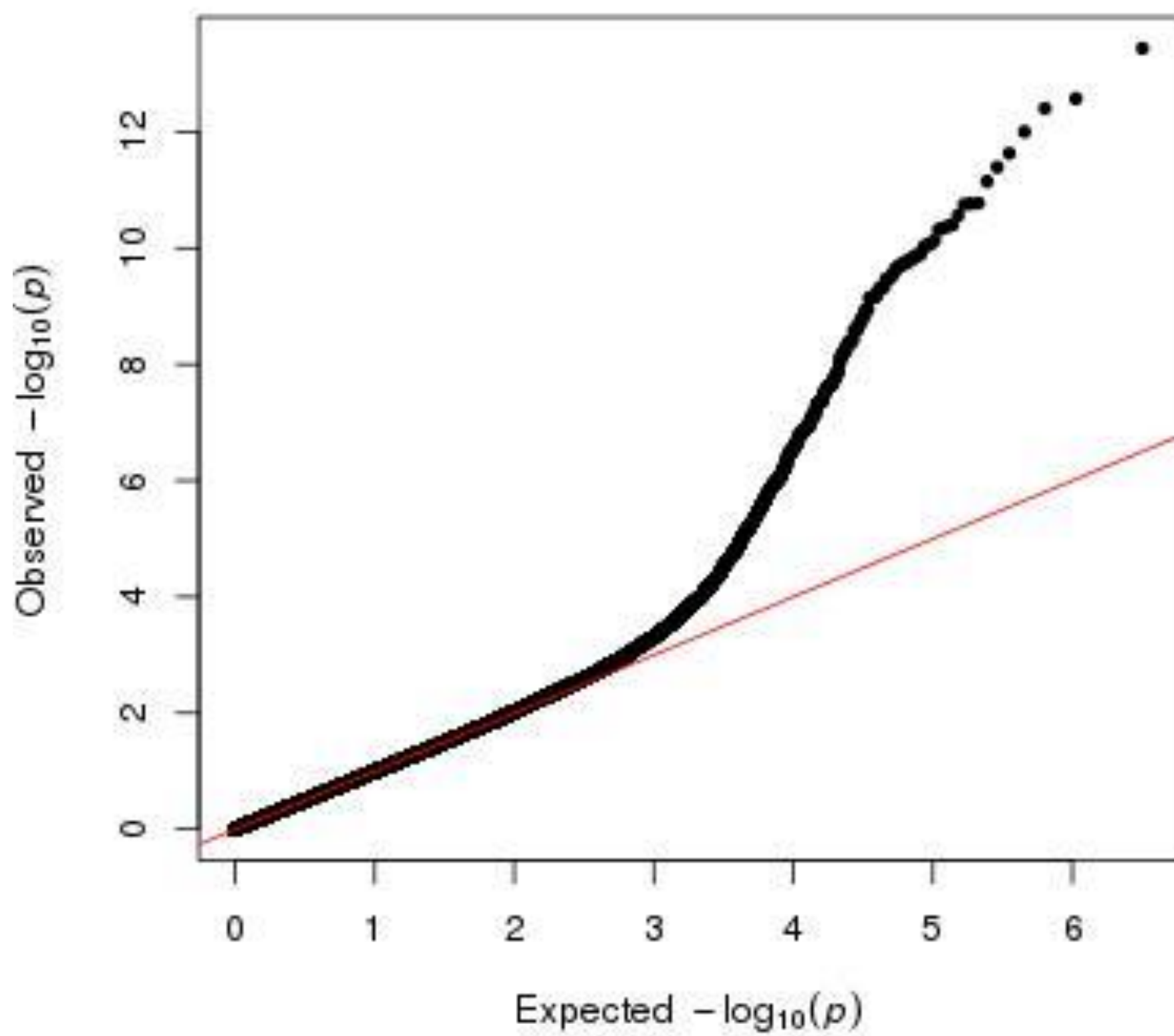

Supplementary figure 1. Q-Q plot of the genome-wide association analysis about eGFR levels

Supplement: Supplementary file 1 — Supplemantary figure 1 [file 41387_2024_316_MOESM1_ESM.pdf]
